# Nanoparticulated Bimodal Contrast Agent for Ultra-High-Field Magnetic Resonance Imaging and Spectral X-ray Computed Tomography

**DOI:** 10.1021/acs.inorgchem.4c01114

**Published:** 2024-05-29

**Authors:** Daniel González-Mancebo, Ana Isabel Becerro, Carlos Caro, Elisabet Gómez-González, María Luisa García-Martín, Manuel Ocaña

**Affiliations:** † Instituto de Ciencia de Materiales de Sevilla (CSIC-US), c/Américo Vespucio, 49, Seville 41092, Spain; ‡ Biomedical Magnetic Resonance Laboratory-BMRL, Andalusian Public Foundation Progress and Health-FPS, Seville 41092, Spain; § Instituto de Investigación Biomédica de Málaga y Plataforma en Nanomedicina − IBIMA Plataforma BIONAND, Málaga 29590, Spain; ∥ CIBER-BBN, ISCIII,Monforte de Lemos 3-5. Pabellón 11. Planta 0, Madrid 28029,Spain

## Abstract

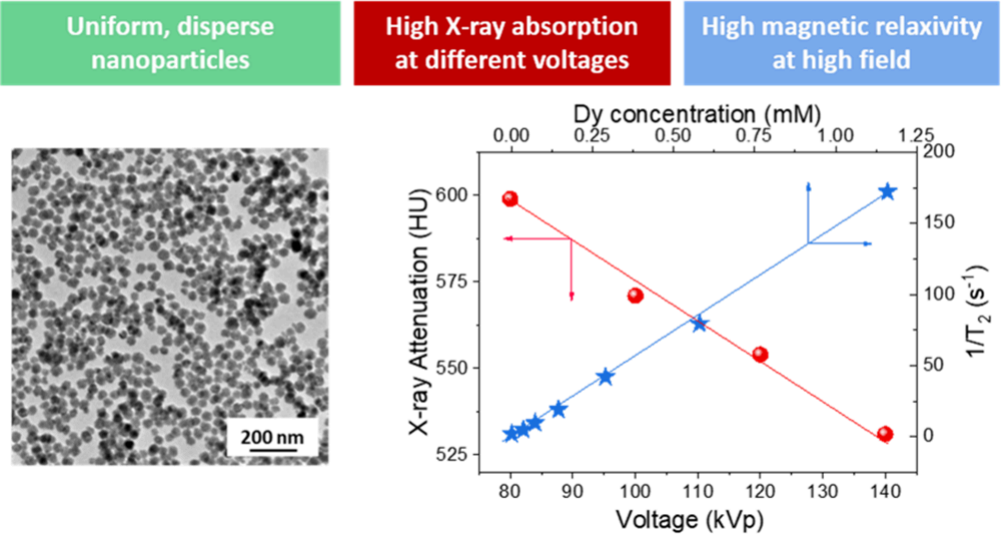

Bimodal medical imaging based on magnetic resonance imaging (MRI) and computed tomography (CT) is a well-known strategy to increase the diagnostic accuracy. The most recent advances in MRI and CT instrumentation are related to the use of ultra-high magnetic fields (UHF-MRI) and different working voltages (spectral CT), respectively. Such advances require the parallel development of bimodal contrast agents (CAs) that are efficient under new instrumental conditions. In this work, we have synthesized, through a precipitation reaction from a glycerol solution of the precursors, uniform barium dysprosium fluoride nanospheres with a cubic fluorite structure, whose size was found to depend on the Ba/(Ba + Dy) ratio of the starting solution. Moreover, irrespective of the starting Ba/(Ba + Dy) ratio, the experimental Ba/(Ba + Dy) values were always lower than those used in the starting solutions. This result was assigned to lower precipitation kinetics of barium fluoride compared to dysprosium fluoride, as inferred from the detailed analysis of the effect of reaction time on the chemical composition of the precipitates. A sample composed of 34 nm nanospheres with a Ba_0.51_Dy_0.49_F_2.49_ stoichiometry showed a transversal relaxivity (*r*_2_) value of 147.11 mM^–1^·s^–1^ at 9.4 T and gave a high negative contrast in the phantom image. Likewise, it produced high X-ray attenuation in a large range of working voltages (from 80 to 140 kVp), which can be attributed to the presence of different K-edge values and high Z elements (Ba and Dy) in the nanospheres. Finally, these nanospheres showed negligible cytotoxicity for different biocompatibility tests. Taken together, these results show that the reported nanoparticles are excellent candidates for UHF-MRI/spectral CT bimodal imaging CAs.

## Abstract 2

Recent advances in clinical imaging instrumentation are related to the use of different working voltages in computed tomography and of ultra-high magnetic fields in magnetic resonance imaging. Such advances, intended to lead to a more precise diagnosis, require the development of contrast agents that are efficient under the new instrumental conditions. This report describes the synthesis and detailed characterization of a carefully designed nanoprobe with excellent properties as a contrast agent for both advanced imaging techniques.

## 1 Introduction

Noninvasive diagnostic imaging techniques, such as magnetic resonance imaging (MRI) and X-ray computed tomography (CT), are among the most extensively utilized clinical imaging modalities due to the advantages of availability, facile image processing, no tissue damage, and painlessness to patients.^1,2^ They allow one to examine a living organism to help diagnose diseases at preclinical and clinical stages. MRI provides extremely detailed images of soft tissues, while CT is particularly effective for imaging bones and lungs. Their combination has been proposed as a promising strategy to provide maximum information using complementary morphologic and/or functional evidence.^3,4^ However, because of the low specificity of both imaging techniques in certain cases, such as solid tumors, the use of CAs becomes necessary to improve diagnosis accuracy. In this context, the development of efficacious bimodal CAs will enable sequential MRI/CT imaging with just one administration, thereby enhancing patient safety.^5,6^

Contrast on MRI is mostly based on the different relaxation rates of protons in the water molecules of the body after the application of a radiofrequency current under the effect of a strong magnet. Clinical MRI is currently conducted with magnetic fields of 1.5–3 T, and gadolinium complexes are widely used as CAs. The use of ultra-high magnetic fields (UHF, > 7 T) increases the signal-to-noise ratio and thus improves imaging quality.^7^ Adoption of high-field imaging constitutes the main trend in future MRI development. Implementation of UHF-MRI requires, however, advances on many fronts, from the technological side^8,9^ to the scientific one. The latter implies the design of new CAs that are more effective at UHF than the gadolinium complexes.^10^ Among them, Dy^3+^-based inorganic NPs are shown as good alternatives due to the high magnetic moment of Dy^3+^, which directly influences the relaxation capacity of the CA.^10−14^ In addition, the use of inorganic NPs is advantageous over gadolinium complexes because, by controlling their size and surface, it is possible to modify their circulation times in the organism and deliver them specifically to the organs of interest, thus reducing the dose required.^15^

On the other hand, CT is based on the different capacities of living tissues to attenuate an X-ray beam. In contrast to conventional X-ray images, CT provides cross-sectional imaging that, as for MRI, can be digitally “stacked” together to form a three-dimensional (3D) image of the region of interest.^16^ In spite of this advantage, it is still difficult to distinguish subtle changes in soft tissues because most of them have similar CT numbers, ranging from 0 to 50 Hounsfield units (HU). Consequently, exogenous CT CAs are required for better delineation of regions of interest.^17,18^ CT CAs used in clinics nowadays are barium salts and iodine complexes, with high Z- and K-edge values, which contributes to a high X-ray attenuation and therefore to a good image contrast. Recent studies have proposed the use of barium and lanthanide-based inorganic nanoparticles (NPs) as CT CAs due to the high Z and high K-edge values of these elements^19,20^ as well as the above-described advantages of inorganic NPs versus salts and complexes. Advances in CT instrumentation can further improve material differentiation by using polychromatic (different voltages) X-ray beams.^21^ The technique, known as spectral CT, has proven utility in providing additional information regarding tissue composition in the musculoskeletal setting, artifact reduction, and image optimization.^22,23^ If different voltages are used in the CT examination, then it is important that the CT CA operates efficiently at any of those voltages. For this purpose, it is necessary to combine, in the same material, elements with high *Z* values and with values of their absorption K-edge close to the energy maxima of the incident X-rays. For example, barium and ytterbium combined in a single nanoparticle were recently shown to maintain high X-ray attenuation at different operating voltages thanks to the great difference in K-edge values between both high-Z elements.^24^ Following this approach, we hypothesize that Ba,Dy-based fluoride NPs must also be useful CAs for spectral CT given the still significant difference in K-edge values between both high-Z elements. In addition, the presence of Dy^3+^ should confer the nanoprobe with contrast capacity for UHF-MRI due to the high magnetic moment of this ion, as commented on above. The chemical combination will therefore produce a bimodal CA for spectral CT and UHF-MRI, which will help to increase diagnostic accuracy through the use of bimodal imaging with the use of a single CA.

Herein, we reported on the synthesis of Ba,Dy-based fluoride nanospheres by homogeneous precipitation in the polyol medium and their characterization by transmission electron microscopy (TEM), energy-dispersive X-ray analysis (EDX), X-ray diffraction, and Fourier transform infrared spectroscopy (FTIR). A detailed analysis of the effects of the Ba/Dy ratio in the starting solutions and of the reaction time on the NPs composition and morphology is also shown. Dispersibility and cytotoxicity were analyzed to evaluate the potential application of such NPs in nanomedicine. Finally, the magnetic relaxivity of the NPs at UHF and their attenuation of multienergy X-rays, generated at different working voltages, were measured and analyzed in order to assess their suitability as bimodal CAs for UHF-MRI and spectral CT.

## 2 Experimental Section

### 2.1 Materials

Dysprosium acetylacetonate (Dy(acac)_3_, Sigma-Aldrich, 99.9%), barium nitrate (Ba(NO_3_)_2_, Sigma-Aldrich, ≥99%), and 1-butyl-3-methylimidazolium tetrafluoroborate ([BMIM]BF_4_, Sigma-Aldrich, ≥97%) were used as NP precursors, while glycerol (Sigma-Aldrich) was used as the solvent for the synthesis reaction.

### 2.2 Synthesis of the NPs

Barium dysprosium NPs were synthesized according to the following reaction: Dy(acac)_3_ and Ba(NO_3_)_2_, with nominal Ba/(Ba + Dy) ratios of 0.50 and 0.75, respectively, were dispersed in glycerol (6.0 mL) with magnetic stirring at 80 °C for 3 h. Total cations concentrations were kept at 0.067 M. After cooling down, 205.5 μL of [BMIM]BF_4_ (0.55 M) was added with magnetic stirring for 3 min at room temperature to favor homogenization. The resulting dispersion was transferred to a tightly closed Teflon test tube and heated for 20 h in an oven preheated at 120 °C. Different reaction times were also tested. After cooling, the dispersion was washed twice with ethanol and once with distilled water using a centrifuge and left in distilled water. A portion of the suspensions was dried at 60 °C for further characterization of the resulting powder.

### 2.3 Morphological, Compositional, and Structural Characterization Techniques

The samples were studied under TEM (JEOL2100Plus, 200 kV) to analyze their shape and size. Their size distribution and mean particle size were obtained from the histogram plotted with the diameters of about 100 particles measured using the free ImageJ software. The experimental Ba/(Ba + Dy) ratio was obtained from the quantification of EDX spectra as well as from ion coupling plasma (ICP) measurements (iCAP 7200 ICP-OES Duo equipment). EDX mappings were recorded to determine the distributions of Ba and Dy elements throughout the nanoparticle. The crystal phase of the precipitates was analyzed by X-ray diffraction using a Panalytical X'PERT PRO equipped with an X-Celerator detector using 300 s of counting time. Fourier transform infrared spectroscopy (FTIR, JASCO FT/IR) was used to analyze the presence of adsorbed molecules on the surface of the NPs. FTIR spectra were recorded in pellets of NPs diluted in KBr. The colloidal stability of the NPs suspended in water (0.5 mg·mL^–1^) was studied via dynamic light scattering (DLS) measurements (Zetasizer Nano-ZS90 Malvern). The DLS curves of the suspension were recorded at different times without removing the suspension from the equipment. Photographs of a 5 mg·mL^–1^ suspension were taken with the CCD camera of a mobile phone (ISO 800, 1/50 s).

### 2.4 Magnetic Relaxivity

The transversal relaxation times (*T*_2_) of aqueous suspensions of the NPs containing different Dy^3+^ concentrations (from 0.0 up to 1.2 mM) were evaluated at conventional (1.44 T) and ultra-high (9.4 T) magnetic fields using the Carl–Purcell–Meiboom–Gill (CPMG) pulse sequence. Measurements at 1.44 T were carried out in a Bruker Minispec (TD-NMR) at 37 °C, while a Bruker Biospec MRI system was used for measurements at 9.4 T at 25 °C. The transversal relaxivity values (*r*_2_) were obtained from the slope of the linear fit of 1/*T*_2_ versus Dy concentration (in mM units).

### 2.5 X-ray Attenuation

X-ray attenuation was measured in aqueous suspensions containing different concentrations of the contrast agent (from 0.0 up to 20.0 mg·mL^–1^) using a Zeiss Xradia 610 Versa 3D X-ray microscope (XRM) equipped with a tungsten anode. Solutions of Iohexol (a commercial iodinated CT CA) were also tested at the same concentrations for comparison. The recording procedure was as follows: 1.0 mL of the CA suspension and 1.0 mL of Milli-Q water, the latter for calibration, were placed in separate Eppendorf tubes. They were then irradiated with 123 μA current and different voltages between 80 and 140 kVp for 0.1 s. The images were collected with a 0.4× objective using no filter (pixel size of 213 μm). The images were reconstructed with the Reconstructor Scout and Scan 16.1.13.038 software using 801 projections. The resulting images were analyzed with free ImageJ software using a 0.5 cm radius for the spherical volume. The final images were calibrated using the average intensity value of water and air to calculate the X-ray attenuation values in Hounsfield units (HU), considering that water attenuation is 0 HU and air attenuation is −1000 HU.

### 2.6 Cytotoxicity

Mitochondrial activity, cell morphology, and necrotic/late apoptotic cells were analyzed using HFF-1 human foreskin fibroblasts by means of MTT and life-dead assays as described elsewhere.^13^

## 3 Results

### 3.1 Synthesis and Characterization of NPs

**Figure 1:**
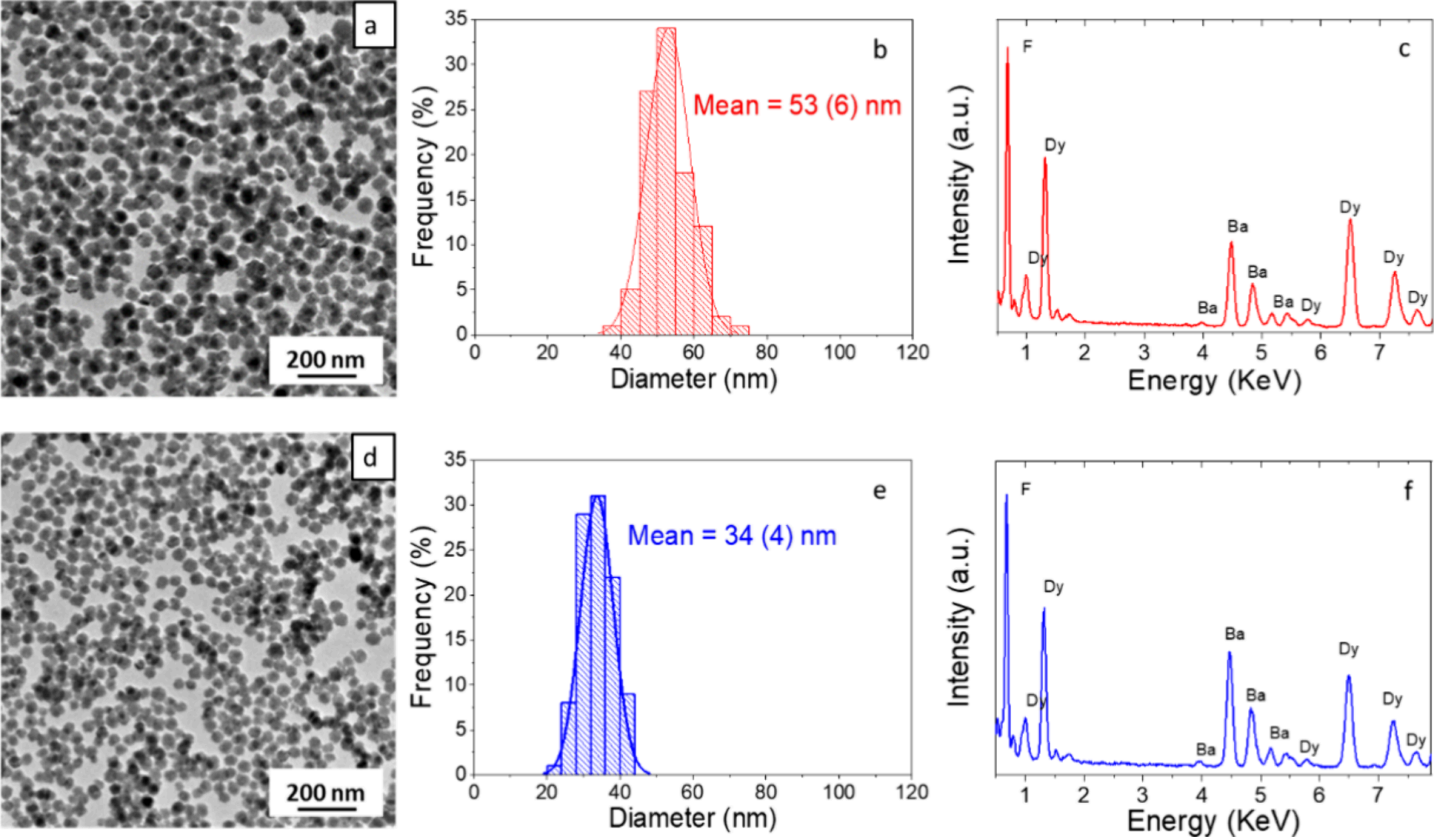
TEM micrographs (a, d), size histograms (b, e), and EDX spectra (c, f) of barium dysprosium fluoride NPs synthesized using nominal Ba/(Ba + Dy) ratios = 0.50 (top, Ba40Dy60 NPs) and 0.75 (bottom, Ba51Dy49 NPs).

It is well-known that uniform particles can be obtained by precipitation through a slow and controlled release of the precipitating anions or cations in the reaction medium.^25^ In this work, fluoride-based NPs containing barium and dysprosium were synthesized using homogeneous precipitation in glycerol through a controlled release of fluoride anions provided by the use of [BMIM](BF_4_) ionic liquid.^14^ Glycerol was selected as the solvent because polyols may act not only as solvents but also as capping agents, thus limiting particle growth, which helps to control particle size.^26^ This experimental approach results in hydrophilic NPs, which is an interesting advantage with respect to synthesis methods of fluoride-based NPs based on the use of oleic acid,^27,28^ which need a second synthesis step to make the NPs water-dispersible and useful for bioapplications. Given that Ba and Dy are expected to attenuate X-ray beams of different energies due to their different K-edge values, as described in the Introduction of this work, our goal was to synthesize NPs with the same molar content of both cations. With this aim in mind, we prepared a glycerol solution of [BMIM](BF_4_), Ba(NO_3_)_2_, and Dy(acac)_3_, with a 0.50 Ba/(Ba + Dy) molar ratio. Figure 1a shows the TEM micrograph of the precipitate obtained after aging such a solution at 120 °C for 20 h. Spherical NPs with 53 (6) nm mean diameter, as inferred from the histogram of Figure 1b, were observed. The EDX spectrum of the sample (Figure 1c) showed exclusively bands corresponding to Ba, Dy, and F, which demonstrated the purity of the precipitate. Quantification of Ba and Dy by ICP gave a Ba/(Ba + Dy) ratio of 0.40 (Ba_0.4_Dy_0.6_F_2.6_ stoichiometry), which is lower than the pursued composition likely due to a lower precipitation rate of Ba fluoride compared to that of Dy fluoride. This sample will be called Ba40Dy60 from now on. We then increased the nominal Ba/(Ba + Dy) ratio up to 0.75 and obtained uniform, spherical NPs (Figure 1d) with 34 (4) nm mean diameter (Figure 1e) and high purity (Figure 1f). The experimental Ba/(Ba + Dy) ratio obtained from ICP was 0.51 (Ba_0.51_Dy_0.49_F_2.49_ stoichiometry), which is very close to the pursued composition. This sample will be called Ba51Dy49 from now on.

**Figure 2:**
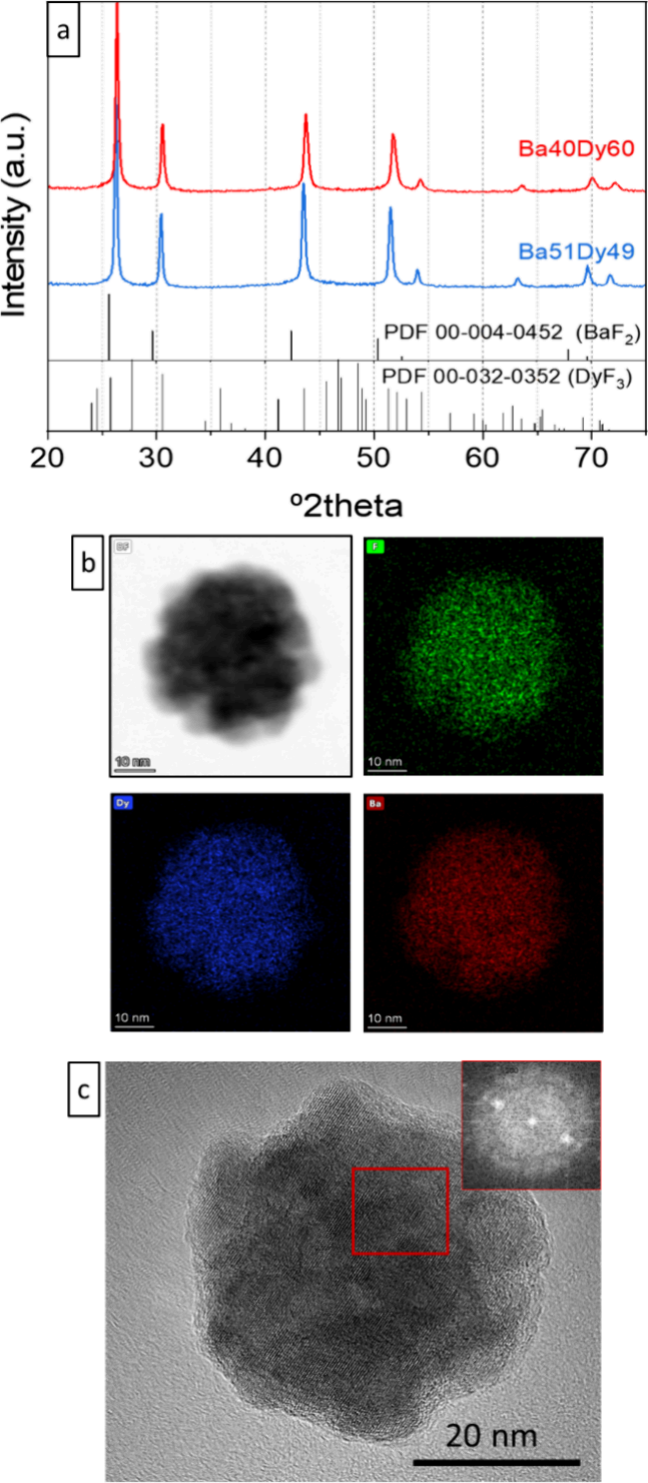
(a) XRD patterns of Ba40Dy60 and Ba51Dy49 samples. Powder diffraction files of cubic BaF_2_ and orthorhombic DyF_3_ are shown at the bottom. (b, c) EDX mappings and HRTEM image of a single Ba51Dy49 NP.

The XRD patterns of the Ba40Dy60 and Ba51Dy49 samples (Figure 2a) showed exclusively reflections compatible with the fluorite-type crystal structure of BaF_2_ (cubic, *Fm*3̅*m*, PDF 00-004-0452), while no reflection corresponding to DyF_3_ (orthorhombic, *Pnma*, PDF 00-032-0352) was observed in the patterns. Compared with the BaF_2_ pattern, the reflections of both samples were shifted toward higher 2θ values, the shift increasing with decreasing the Ba/(Ba + Dy) ratio. Accordingly, the cubic unit cell parameters obtained from the LeBail analysis of the Ba51Dy49 and Ba40Dy60 patterns using space group *Fm*3̅*m* were lower (5.8961 (6) and 5.8782 (6) Å, respectively) than that reported for BaF_2_ (6.2001 Å) (PDF 00-004-0452). This finding suggests the formation of a solid solution by replacement of Ba^2+^ ions (ionic radius in VIII coordination of 1.420 Å) with smaller Dy^3+^ ions (ionic radius in VIII coordination of 1.027 Å) in the BaF_2_ crystal structure, in good agreement with the observations for BaLuF_5_-based nanoparticles.^29^ The homogeneous distribution of Ba and Dy in the crystal structure, with the absence of element segregation, observed in the EDX mappings recorded on the NPs with the pursued composition (sample Ba51Dy49), also agreed with the formation of a solid solution (Figure 2b). Finally, the HRTEM micrograph of a single Ba51Dy49 NP (Figure 2c), showed lattice fringes running all through the NP, which suggests a single crystal character. The corresponding lattice spacing was 2.9 Å, which is compatible with the (200) interplanar distance of the Ba_0.51_Dy_0.49_F_2.49_ cubic structure.

**Figure 3:**
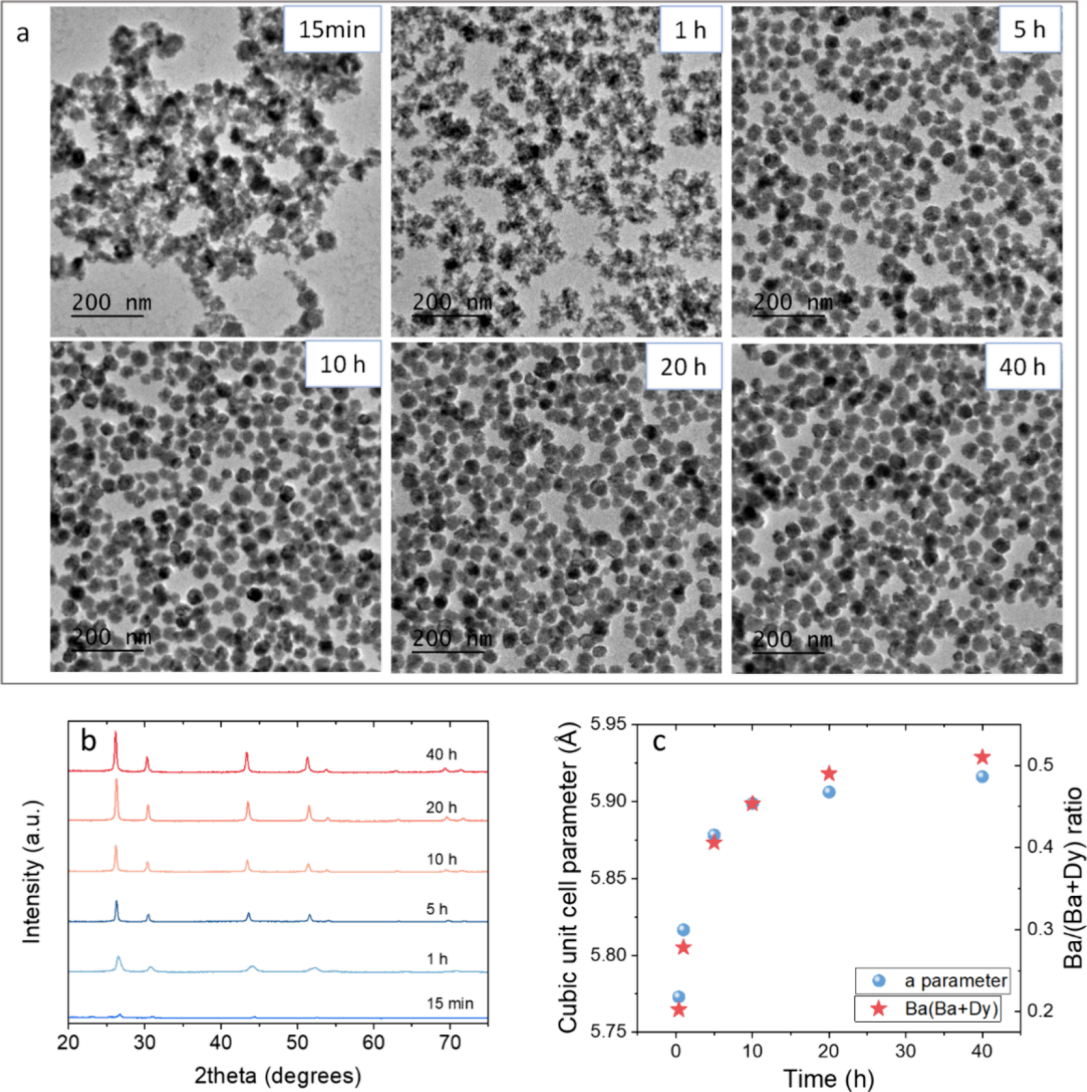
(a) TEM micrographs of the precipitates obtained in the conditions used for the synthesis of Ba51Dy49 NPs but using different aging times (labeled). (b) XRD patterns of the corresponding precipitates. (c) Unit cell parameters and Ba/(Ba + Dy) ratio of the precipitates versus aging time.

In order to shed light on the reasons for the mismatch between the nominal and experimental Ba/(Ba + Dy) ratios observed in the synthesis experiments described at the beginning of this section, we carried out the synthesis of the Ba51Dy49 NPs using reaction times shorter and longer than 20 h and keeping the rest of the experimental parameters fixed. The TEM micrographs of the corresponding precipitates are shown in Figure 3a. The micrograph of the sample obtained after 15 min showed an amorphous precipitate constituted by aggregated particles with an undefined shape. After 1 h of reaction, the precipitate showed poorly defined, porous particles, which still appeared aggregated to each other. When the reaction was left to go for 5 and 10 h, the products looked very similar to each other and to that of the Ba51Dy49 sample (20 h reaction), showing well-defined spherical NPs of around 35 nm diameter (Figure S1). No significant changes were observed after 40 h of reaction either. The XRD patterns of all samples are displayed in Figure 3b. The pattern of the 15 min sample showed poorly defined reflections with very low intensity, while increasing the reaction time gave rise to higher intensity reflections corresponding, in all cases, to the cubic fluorite-type phase. It is also to be noticed that increasing reaction time from 1 to 5 h resulted in narrower reflections, while their width remained constant for longer times. This result suggests that the maximum crystallinity of the samples was reached after 5 h of reaction, when the NPs got their final shape. Finally, it can be observed that increasing reaction time gave rise to the shift of the reflections toward lower angles, which indicated an increase in the unit cell size. This effect can be better visualized in Figure 3c (blue spheres), which shows the cubic unit cell parameter, obtained from the LeBail analysis of the XRD patterns shown in Figure 3b, versus reaction time. The values showed a sharp increase with increasing reaction time up to 5 h, while a much smoother increase was observed for longer reaction times. The increase in unit cell size with increasing reaction time could be related to the progressive incorporation of Ba^2+^ substituting Dy^3+^ in the cubic fluorite structure (Ba^2+^ ionic radius in VIII coordination = 1.420 Å to be compared to 1.027 Å for Dy^3+^). To check this hypothesis, we plotted the Ba/(Ba + Dy) ratio of the precipitates in the same graph (Figure 3c, red stars), observing a parallel trend that confirms our hypothesis. We can therefore conclude that the precipitation rate of barium fluoride is lower than that of dysprosium fluoride, and this must be the reason for the mismatch between the nominal and experimental Ba/(Ba + Dy) ratio observed in the synthesis experiments.

### 3.2 Colloidal Stability and Cytotoxicity

**Figure 4:**
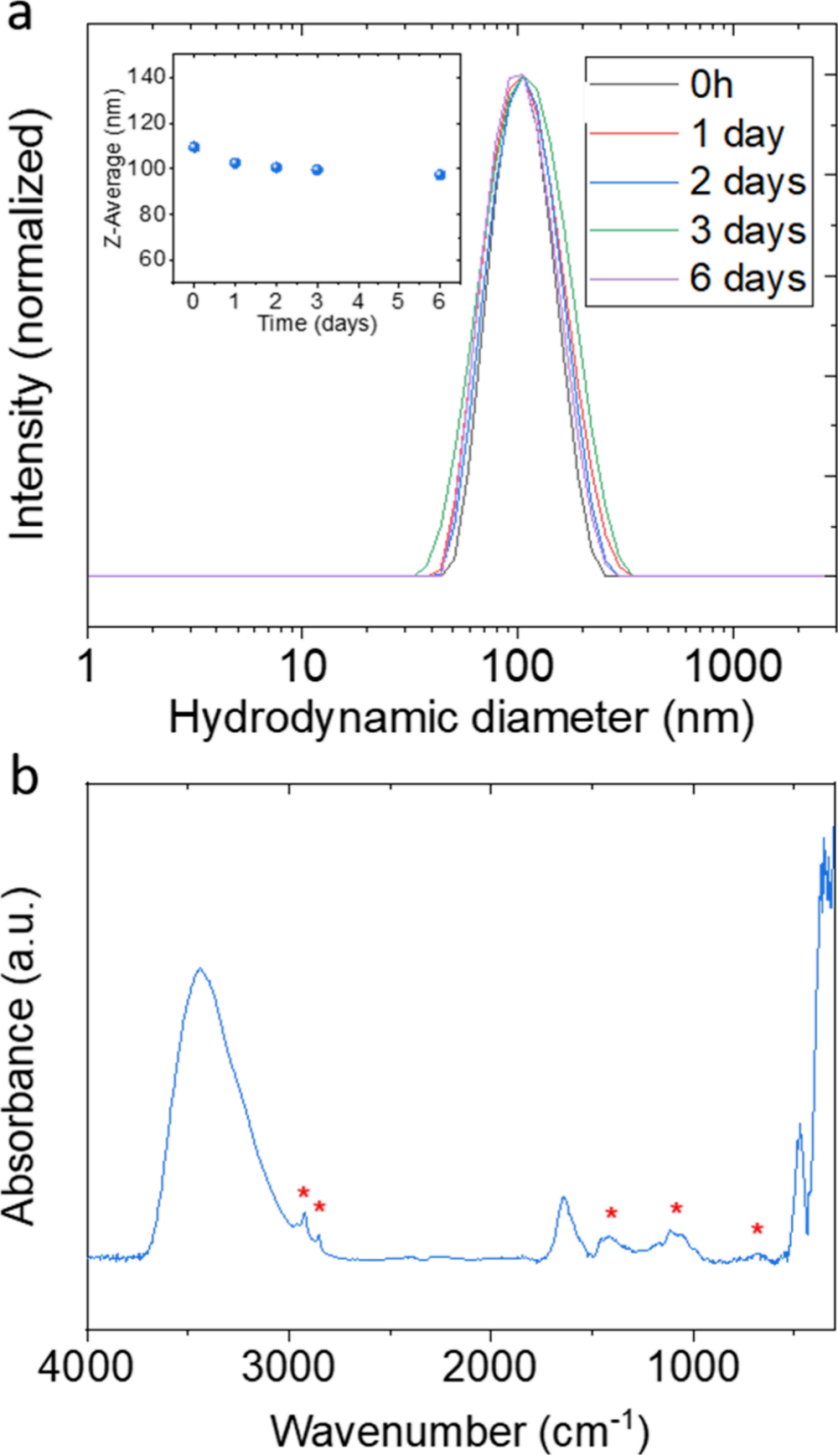
(a) DLS intensity curves of the Ba51Dy49 NPs suspended in water after different periods at rest. The inset is the *z*-average obtained from the corresponding DLS curves. (b) FTIR spectrum of the Ba51Dy49 NPs. Asterisks indicate bands corresponding to the organic groups.

It is well-known that nanoparticles to be used in biomedical applications should fulfill several requisites among which colloidal stability in aqueous media and absence of toxicity are essential.^30^Figure S2 shows the photographs of the Ba51Dy49 NPs suspended in water taken at different times at rest. The suspensions did not show any signs of aggregation or sedimentation under the naked eye. To further evaluate the colloidal stability of the suspension, DLS curves were recorded with time, while the suspension was kept at rest inside the DLS equipment (Figure 4a). The curves were similar to each other, with no significant changes in position or width up to 6 days, with the seventh day being unstable for DLS measurement. The sizes (*z*-average) obtained from the curves were close to 100 nm in all cases (inset of Figure 4a). The DLS results confirm the colloidal stability of the NP suspension for 6 days. The FTIR spectrum (Figure 4b) showed a set of narrow bands located at <500 cm^–1^, characteristic of fluoride matrices,^31^ and two bands at 3440 and 1650 cm^–1^ that indicate the presence of adsorbed water molecules on the surface of the NP. In addition, the lower intensity bands, marked with asterisks in the figure, correspond to organic groups of glycerol^32^ and suggest the presence of adsorbed solvent molecules on the surface of the NPs. Such molecules could be responsible for the good dispersibility shown by the NPs.

**Figure 5:**
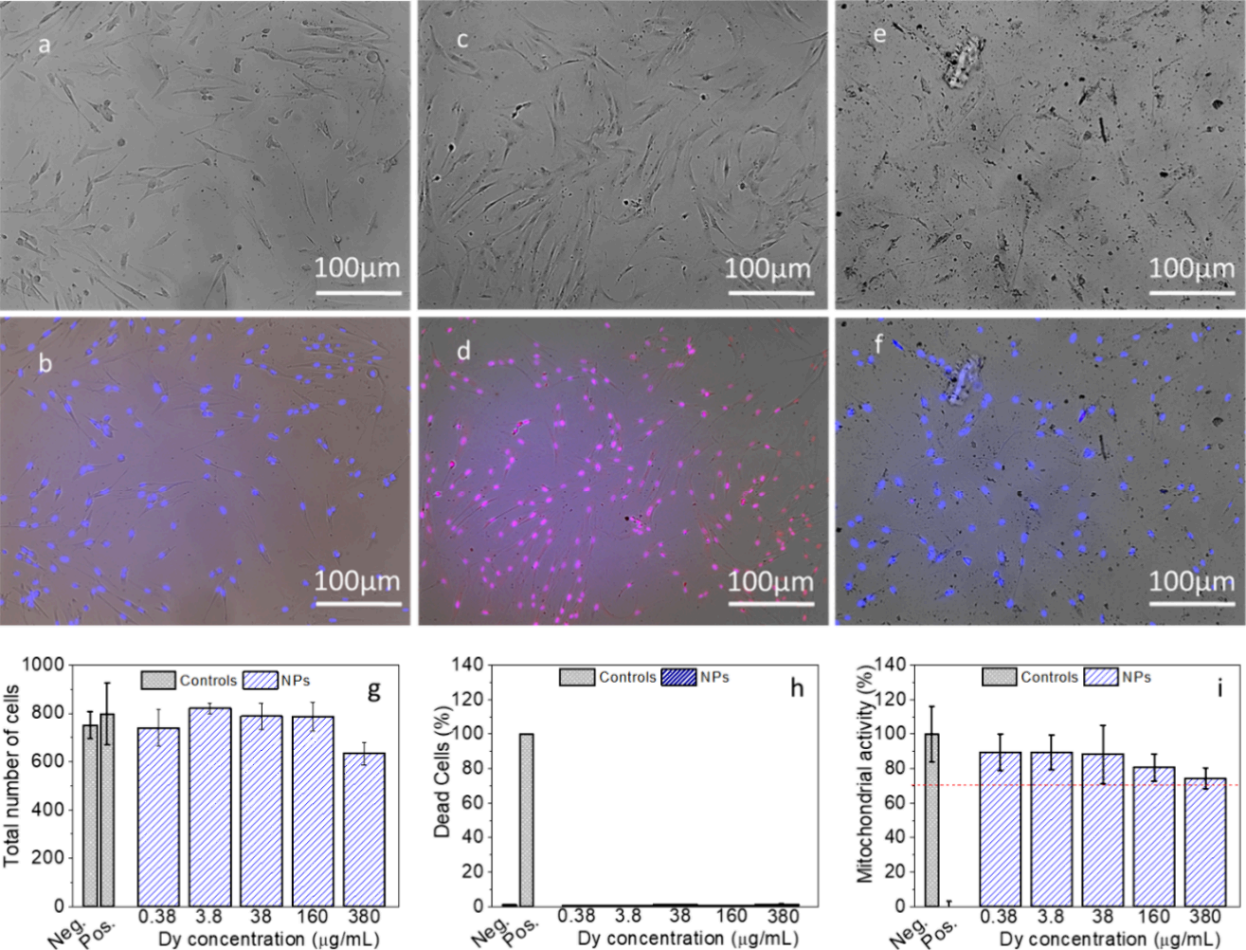
Bright field optical microscopy images of HFF-1 fibroblasts (top) and images resulting from the merge (bottom) of bright field, Hoechst 33342 (blue), and TO-PRO-3 iodine (red) images: (a, b) negative control, (c, d) positive control, and (e, f) cells exposed to 380 μg·mL^–1^ for Ba51Dy49 NPs. (g) Total number of cells per well after exposure to increasing concentration of NPs. (h) Percentage of dead cells after exposure to increasing concentration of Ba51Dy49 NPs. (i) MTT assay of cells exposed to increasing concentration of Ba51Dy49 NPs.

The analysis of cytotoxicity of Ba51Dy49 NPs to evaluate their potential application in biomedicine was carried out through different studies, namely, cell morphology, induction of necrotic/late apoptotic cells, and mitochondrial activity of HFF-1 human foreskin fibroblasts incubated with the NPs (Figure 5). The microscopy images of the cells incubated with up to 380 μg Dy·mL^–1^ (Figure 5a–f), more than 3 times higher than those typically used in cell culture assessments,^33−35^ showed no notable change in their morphology. Likewise, the total number of cells did not vary significantly, even at the highest concentration of NPs studied (Figure 5g), indicating the absence of necrosis. Moreover, this NP concentration did not generate a relevant increase in cell death by apoptosis (Figure 5h) nor did it decrease the mitochondrial activity below 75% relative to that of the negative control (Figure 5i). These results clearly indicate negligible cytotoxicity under the conditions tested and, therefore, the suitability of the Ba51Dy49 NPs for use in nanomedicine.

### 3.3 Magnetic Resonance Relaxivity at Low and Ultra-High Magnetic Fields

**Figure 6:**
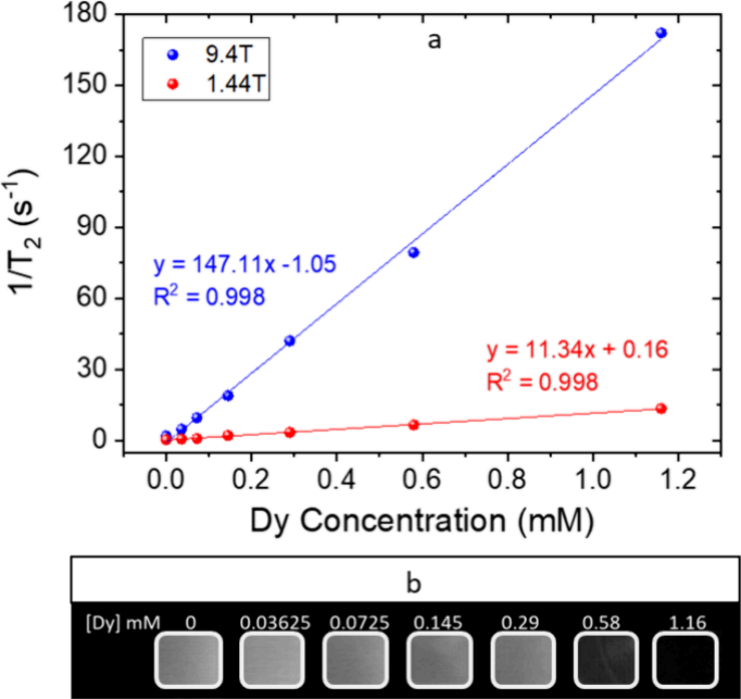
(a) 1/*T*_2_ values obtained in aqueous suspensions of Ba51Dy49 NPs at 9.4 and 1.44 T versus Dy concentration of the suspensions. The slopes of the linear fits provide the transversal relaxivity. (b) *T*_2_-weighted MR images of the Ba51Dy49 NPs in water at 9.4 T with increasing concentrations.

The high magnetic moment (∼10.6 μB) and short electronic relaxation time of Dy^3+^ ions (∼10^–3^ s) are expected to confer the Ba51Dy49 NPs with a high transversal magnetic relaxivity (*r*_2_).^36^ The transverse proton relaxation times (*T*_2_) of aqueous suspensions containing different concentrations of NPs were measured under a conventional (1.44 T) and an ultra-high (9.4 T) magnetic field. The values were plotted as 1/*T*_2_ versus Dy^3+^ concentration in Figure 6a, and the data were successfully fitted to a line in both cases, indicating the absence of aggregation in the concentration range tested and in agreement with the stability observed by DLS. The slopes of the linear fits gave relaxivity values (*r*_2_) of 11.34 and 147.11 for the conventional and ultra-high magnetic fields, respectively. The *r*_2_ value observed at an ultra-high magnetic field (147.11 mM^–1^·s^–1^) was larger than that reported in the literature for other Dy^3+^-based NPs measured at the same field of 9.4 T (91.4 and 101 mM^–1^·s^–1^),^12,37^ which would result in a higher contrast of the MRI images. The observed increase in relaxivity with increasing magnetic field strength can be explained on the basis of the paramagnetic relaxation enhancement (PRE), which is the interaction of lanthanides with the proton spins.^36^ The PRE can be expressed as the sum of three components, namely, contact, dipolar, and Curie terms, the latter being predominant for lanthanides other than Gd^3+^. The Curie component depends, among other factors, on the square of the applied magnetic field strength, and it would be responsible for the observed increment of *r*_2_ at ultra-high field compared with the value observed under a conventional field.^38^ Finally, Figure 6b shows the magnetic resonance phantom images obtained at 9.4 T in aqueous suspensions with different concentrations of Ba51Dy49 NPs. It can be seen that the image darkens with increasing NP concentration, confirming the suitability of such NPs as negative (or *T*_2_) MRI CAs.

### 3.4 X-ray Attenuation Properties

**Figure 7:**
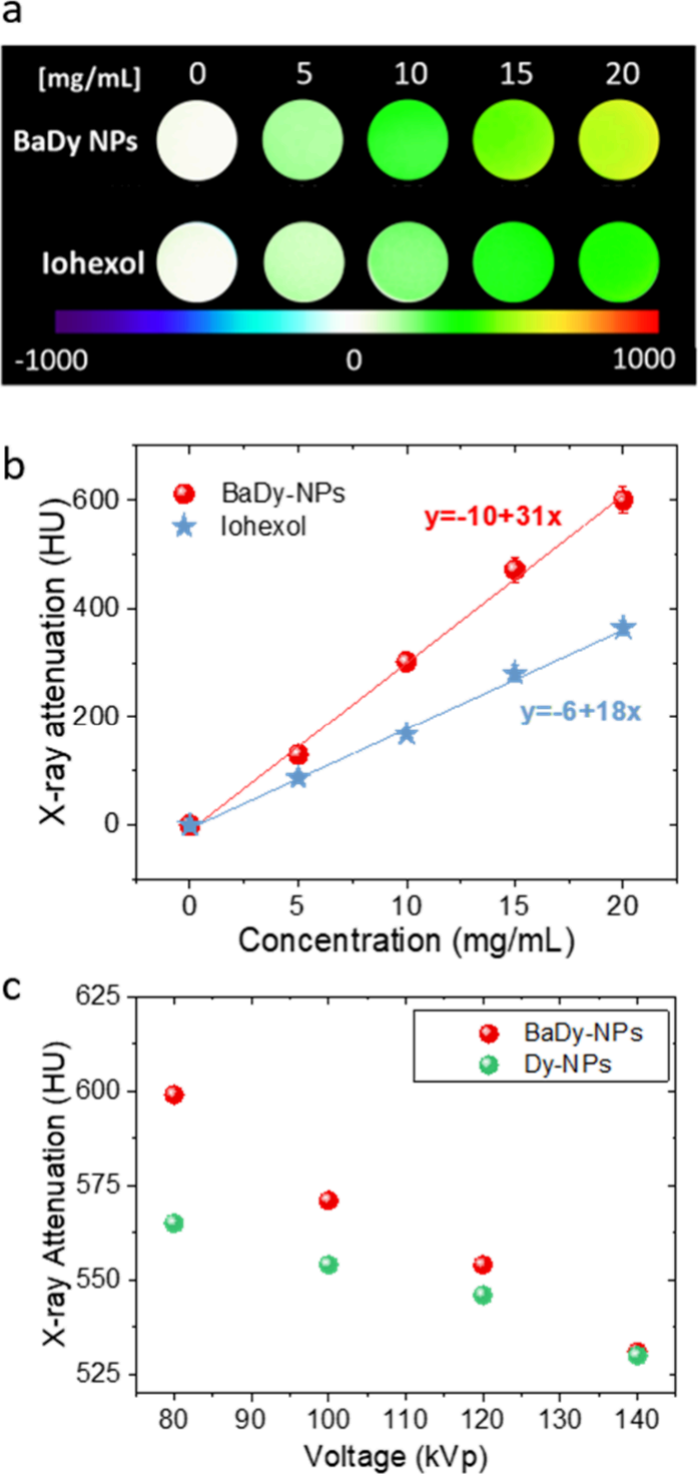
(a) CT phantom images, recorded at 80 kVp, of aqueous suspensions containing increasing amounts of Ba51Dy49 NPs (top) and Iohexol (bottom). (b) X-ray attenuation at 80 kVp, in Hounsfield Units (HU), of aqueous suspensions containing Ba51Dy49 NPs and Iohexol, versus suspension concentration. (c) X-ray attenuation, in HU, of aqueous suspensions containing 20.0 mg·mL^–1^ for Ba51Dy49 and DyF_3_ NPs, versus working voltage.

The X-ray attenuation capacity of the Ba51Dy49 NPs dispersed in water was first measured at 80 kVp as a function of the suspension concentration. The phantom images in Figure 7a show a higher contrast for Ba51Dy49 NPs than for Iohexol, a commercial iodinated CT contrast agent, at any concentration. The higher X-ray attenuation can be better inferred from the plot of X-ray attenuation in Hounsfield units obtained from the phantom images versus the concentration of the CAs in the aqueous suspension (Figure 7b). For both suspensions, a linear increase in attenuation is observed with increasing suspension concentration. The slope of the linear fits was 31 for Ba51Dy49 NPs and 18 for Iohexol, which demonstrates the higher X-ray attenuation capacity of the NPs compared with the commercial agent, which results in the need of a lower dosis to get the same contrast. Thus, to get a change in X-ray attenuation of 100 HU, we would need 4.9 mg of Iohexol·mL^–1^ while only 2.9 mg of NPs·mL^–1^ (equivalent to 7.3 mM Dy) would be necessary. The observed behavior agrees well with the higher *Z* value of Ba (*Z* = 56) and Dy (*Z* = 66) compared with I (*Z* = 53) as the X-ray attenuation coefficient of a given material depends on *Z*^4^.^17^ Likewise, the X-ray attenuation capacity of these NPs is of the same order as that observed for other lanthanide-based fluoride NPs described in the literature.^19,39^ It can be therefore concluded that the Ba51Dy49 NPs are good candidates as CAs for conventional CT, improving the performance of Iohexol, as they produce the same attenuation with a lower dose, which would be beneficial for the patient. However, it is important to remark that further investigations on the biodistribution of the Ba51Dy49 NPs in the organism are necessary for a suitable comparison with the molecular contrast agent Iohexol.

Second, we measured the ability of Ba51Dy49 NPs to work as a CA in spectral CT. As mentioned in the Introduction, spectral CT refers to the use of energy information from polychromatic X-rays. The capability of Ba51Dy49 NPs as a CA in spectral CT was then tested by measuring the X-ray attenuation of an aqueous suspension containing 20.0 mg·mL^–1^ for Ba51Dy49 NPs at different voltages, from 80 up to 140 kVp, which are typically used in clinics. To demonstrate the effect of the combination of Ba and Dy on the X-ray attenuation at different voltages, DyF_3_ NPs were synthesized using the same conditions as for Ba51Dy49 NPs but in the absence of barium (Figure S3). The attenuation values of suspensions of Ba51Dy49 NPs and DyF_3_ NPs (Figure 7c) decreased in both cases when the potential was increased from 80 to 140 kVp. This is because the X-ray absorption coefficient (μ) is inversely proportional to the incident beam energy (μ = (ρ*Z*^4^)/(*AE*^3^), where ρ is the material density, *Z* is the atomic number, *A* is the atomic mass, and *E* is the X-ray energy).^17^ It can also be observed that the X-ray attenuation values obtained for both suspensions were practically the same at a high voltage (140 kVp). Attenuation at this voltage is mainly due to the effect of Dy present in both samples. The Dy absorption K-edge (53.8 keV) is in close proximity to the maximum energy of the characteristic radiation (56–63 keV) of the X-rays at such voltage (tungsten anode), which maximizes the beam attenuation.^40^ As the voltage decreases, the energy of the applied X-ray beam also decreases, and consequently, the attenuation produced by the DyF_3_ NPs and Ba51Dy49 NPs increased, as observed in Figure 7c. However, a much more pronounced increase in attenuation was observed for Ba51Dy49 NPs than for DyF_3_. This different behavior is due to Ba, whose absorption K-edge (37 keV) is close to the maximum of the radiation emitted at low working voltages (between 40 and 50 keV), which leads to an increase in the X-ray attenuation value in the Ba51Dy49 NPs. In conclusion, the coexistence of Ba and Dy confers the NPs with high X-ray attenuation values at both high and low working voltages, thus making them good candidates as contrast agents for spectral CT.

## 4 Conclusions

Uniform nanospheres of barium dysprosium fluoride with a Ba/(Ba + Dy) molar ratio close to 1 (Ba_0.51_Dy_0.49_F_2.49_) were synthesized following a solvothermal method by homogeneous precipitation from a glycerol solution of the precursor salts. It was necessary to use a Ba/(Ba + Dy) molar ratio = 0.75 instead of 0.50 in the starting solution in order to obtain particles with the pursued Ba–Dy equimolar composition. A detailed study of the reaction as a function of time indicated that the mismatch between the starting and final Ba/(Ba + Dy) ratio was likely due to a lower precipitation kinetics of barium fluoride compared to dysprosium fluoride. The Ba_0.51_Dy_0.49_F_2.49_ nanospheres showed a high magnetic transversal relaxivity value under ultra-high magnetic field and excellent X-ray absorption properties at any working voltage. These properties, combined with their good dispersion in liquid media and remarkable biocompatibility, make barium fluoride nanoparticles an outstanding platform as a novel, bimodal contrast agent for UHF-MRI and spectral CT.

## Supporting Information Available

Size histograms obtained from TEM micrographs of the NPs synthesized at different reaction times using nominal Ba/(Ba + Dy) = 0.75 (Figure S1), photographs of Ba51Dy49 NP suspensions taken after different periods at rest (Figure S2), and TEM micrograph and size histograms of DyF_3_ NPs (Figure S3) (PDF)

## Notes

The data that support the findings of this study are openly available in Digital CSIC repository at https://doi.org/10.20350/digitalCSIC/16267.

## References

[ref1] Khalid H.; Hussain M.; Al Ghamdi M. A.; Khalid T.; Khalid K.; Khan M. A.; Fatima K.; Masood K.; Almotiri S. H.; Farooq M. S.; et al. A Comparative Systematic Literature Review on Knee Bone Reports from MRI, X-Rays and CT Scans Using Deep Learning and Machine Learning Methodologies. Diagnostics 2020, 10, 518 10.3390/diagnostics10080518. 32722605 PMC7460189

[ref2] Junn J. C.; Soderlund K. A.; Glastonbury C. M. Imaging of Head and Neck Cancer With CT, MRI, and US. Seminars in Nuclear Medicine 2021, 51, 3–12. 10.1053/j.semnuclmed.2020.07.005. 33246537

[ref3] Wallyn J.; Anton N.; Mertz D.; Begin-Colin S.; Perton F.; Serra C. A.; Franconi F.; Lemaire L.; Chiper M.; Libouban H.; et al. Magnetite- and Iodine-Containing Nanoemulsion as a Dual Modal Contrast Agent for X-ray/Magnetic Resonance Imaging. ACS Appl. Mater. Interfaces 2019, 11, 403–416. 10.1021/acsami.8b19517. 30541280

[ref4] Ding X.; Hao X.; Fu D.; Zhang M.; Lan T.; Li C.; Huang R.; Zhang Z.; Li Y.; Wang Q.; et al. Gram-scale synthesis of nanotherapeutic agents for CT/T_1_-weighted MRI bimodal imaging guided photothermal therapy. Nano Research 2017, 10, 3124–3135. 10.1007/s12274-017-1530-6.

[ref5] Wahsner J.; Gale E. M.; Rodriguez-Rodriguez A.; Caravan P. Chemistry of MRI Contrast Agents: Current Challenges and New Frontiers. Chem. Rev. 2019, 119, 957–1057. 10.1021/acs.chemrev.8b00363. 30350585 PMC6516866

[ref6] Ashton J. R.; West J. L.; Badea C. T. In vivo small animal micro-CT using nanoparticle contrast agents. Front. Pharmacol. 2015, 6, 256 10.3389/fphar.2015.00256. 26581654 PMC4631946

[ref7] Dumoulin S. O.; Fracasso A.; van der Zwaag W.; Siero J. C. W.; Petridou N. Ultra-high field MRI: Advancing systems neuroscience towards mesoscopic human brain function. Neuroimage 2018, 168, 345–357. 10.1016/j.neuroimage.2017.01.028. 28093360

[ref8] Moser E.; Laistler E.; Schmitt F.; Kontaxis G. Ultra-High Field NMR and MRI - The Role of Magnet Technology to Increase Sensitivity and Specificity. Front. Phys. 2017, 5, 33 10.3389/fphy.2017.00033.

[ref9] Rispoli J. V.; Wilcox M. D.; By S.; Wright S. M.; McDougall M. P. Effects of Coplanar Shielding for High Field MRI. In 38th Annual International Conference of the IEEE-Engineering-in-Medicine-and-Biology-Society (EMBC); IEEE: Orlando, FL; 2016, 6250–6253. 10.1109/EMBC.2016.7592157 PMC5548299 28269680

[ref10] Du H.; Wang Q.; Liang Z.; Li Q.; Li F.; Ling D. Fabrication of magnetic nanoprobes for ultrahigh-field magnetic resonance imaging. Nanoscale 2022, 14, 17483–17499. 10.1039/D2NR04979A. 36413075

[ref11] Das G. K.; Zhang Y.; D’Silva L.; Padmanabhan P.; Heng B. C.; Chye Loo J. S.; Selvan S. T.; Bhakoo K. K.; Yang Tan T. T. Single-Phase Dy_2_O_3_:Tb^3+^ Nanocrystals as Dual-Modal Contrast Agent for High Field Magnetic Resonance and Optical Imaging. Chem. Mater. 2011, 23, 2439–2446. 10.1021/cm2003066.

[ref12] Das G. K.; Johnson N. J.; Cramen J.; Blasiak B.; Latta P.; Tomanek B.; van Veggel F. C. NaDyF_4_ Nanoparticles as T_2_ Contrast Agents for Ultrahigh Field Magnetic Resonance Imaging. J. Phys. Chem. Lett. 2012, 3, 524–529. 10.1021/jz201664h. 26286058

[ref13] Gomez-Gonzalez E.; Caro C.; Garcia-Martin M. L.; Becerro A. I.; Ocana M. Outstanding MRI contrast with dysprosium phosphate nanoparticles of tuneable size. Nanoscale 2022, 14, 11461–11470. 10.1039/D2NR02630A. 35904370

[ref14] González-Mancebo D.; Becerro A. I.; Rojas T. C.; García-Martín M. L.; De la Fuente J. M.; Ocaǹa M. HoF_3_ and DyF_3_ Nanoparticles as Contrast Agents for High-Field Magnetic Resonance Imaging. Part. Part. Syst. Charact. 2017, 34, 1700116 10.1002/ppsc.201700116.

[ref15] Nam J.; Won N.; Bang J.; Jin H.; Park J.; Jung S.; Jung S.; Park Y.; Kim S. Surface engineering of inorganic nanoparticles for imaging and therapy. Adv. Drug Deliv Rev. 2013, 65, 622–648. 10.1016/j.addr.2012.08.015. 22975010

[ref16] Gharieb R. R. Computed-Tomography (CT) Scan; IntechOpen 2022.

[ref17] Lusic H.; Grinstaff M. W. X-ray-computed tomography contrast agents. Chem. Rev. 2013, 113, 1641–1666. 10.1021/cr200358s. 23210836 PMC3878741

[ref18] Jiang Z.; Zhang M.; Li P.; Wang Y.; Fu Q. Nanomaterial-based CT contrast agents and their applications in image-guided therapy. Theranostics 2023, 13, 483–509. 10.7150/thno.79625. 36632234 PMC9830442

[ref19] Sun Y.; Zhu X.; Peng J.; Li F. Core-shell lanthanide upconversion nanophosphors as four-modal probes for tumor angiogenesis imaging. ACS Nano 2013, 7, 11290–11300. 10.1021/nn405082y. 24205939

[ref20] Yan J.; Li B.; Yang P.; Lin J.; Dai Y. Progress in Light-Responsive Lanthanide Nanoparticles toward Deep Tumor Theranostics. Adv. Funct. Mater. 2021, 31, 2104325 10.1002/adfm.202104325.

[ref21] Yeh B. M.; FitzGerald P. F.; Edic P. M.; Lambert J. W.; Colborn R. E.; Marino M. E.; Evans P. M.; Roberts J. C.; Wang Z. J.; Wong M. J.; et al. Opportunities for new CT contrast agents to maximize the diagnostic potential of emerging spectral CT technologies. Adv. Drug Deliv Rev. 2017, 113, 201–222. 10.1016/j.addr.2016.09.001. 27620496 PMC5344792

[ref22] Tsurusaki M.; Sofue K.; Hori M.; Sasaki K.; Ishii K.; Murakami T.; Kudo M.; et al. Dual-energy computed tomography of the liver: uses in clinical practices and applications. Diagnostics 2021, 11, 161 10.3390/diagnostics11020161. 33499201 PMC7912647

[ref23] Wu J. L. Y.; Stordy B. P.; Nguyen L. N. M.; Deutschman C. P.; Chan W. C. W. A proposed mathematical description of in vivo nanoparticle delivery. Adv. Drug Delivery Rev. 2022, 189, 114520 10.1016/j.addr.2022.114520. 36041671

[ref24] Liu Y.; Ai K.; Liu J.; Yuan Q.; He Y.; Lu L. Hybrid BaYbF_5_ nanoparticles: novel binary contrast agent for high-resolution in vivo X-ray computed tomography angiography. Adv. Healthc Mater. 2012, 1, 461–466. 10.1002/adhm.201200028. 23184777

[ref25] Matijevic E. Preparation and properties of uniform size colloids. Chem. Mater. 1993, 5, 412–426. 10.1021/cm00028a004.

[ref26] Feldmann C. Polyol-Mediated Synthesis of Nanoscale Functional Materials. Adv. Funct. Mater. 2003, 13, 101–107. 10.1002/adfm.200390014.

[ref27] Mai H. X.; Zhang Y. W.; Si R.; Yan Z. G.; Sun L. D.; You L. P.; Yan C. H. High-quality sodium rare-earth fluoride nanocrystals: controlled synthesis and optical properties. J. Am. Chem. Soc. 2006, 128, 6426–6436. 10.1021/ja060212h. 16683808

[ref28] Niu W.; Wu S.; Zhang S. A facile and general approach for the multicolor tuning of lanthanide-ion doped NaYF_4_ upconversion nanoparticles within a fixed composition. J. Mater. Chem. 2010, 20, 9113–9117. 10.1039/c0jm01879a.

[ref29] González-Mancebo D.; Becerro A. I.; Cantelar E.; Cussó F.; Briat A.; Boyer D.; Ocaǹa M. Crystal structure, NIR luminescence and X-ray computed tomography of Nd^3+^:Ba0.3Lu0.7F2.7 nanospheres. Dalton Trans. 2017, 46, 6580–6587. 10.1039/C7DT00453B. 28447684

[ref30] Gnach A.; Lipinski T.; Bednarkiewicz A.; Rybka J.; Capobianco J. A. Upconverting nanoparticles: assessing the toxicity. Chem. Soc. Rev. 2015, 44, 1561–1584. 10.1039/C4CS00177J. 25176037

[ref31] Nyquist R. A.; Kagel R. O. Spectra. In Handbook of Infrared and Raman Spectra of Inorganic Compounds and Organic Salts; Academic Press, 1971, 48–495.

[ref32] Glycerin https://webbook.nist.gov/cgi/cbook.cgi?ID=C56815&Type=IR-SPEC&Index=1.

[ref33] Paez-Munoz J. M.; Gamez F.; Fernandez-Afonso Y.; Gallardo R.; Pernia Leal M.; Gutierrez L.; de la Fuente J. M.; Caro C.; Garcia-Martin M. L. Optimization of iron oxide nanoparticles for MRI-guided magnetic hyperthermia tumor therapy: reassessing the role of shape in their magnetocaloric effect. J. Mater. Chem. B 2023, 11, 11110–11120. 10.1039/D3TB01821K. 37947078

[ref34] Garcia-Garcia G.; Caro C.; Fernandez-Alvarez F.; Garcia-Martin M. L.; Arias J. L. Multi-stimuli-responsive chitosan-functionalized magnetite/poly(ε-capro-lactone) nanoparticles as theranostic platforms for combined tumor magnetic resonance imaging and chemotherapy. Nanomedicine-Nanotechnology Biology and Medicine 2023, 52, 102695 10.1016/j.nano.2023.102695. 37394106

[ref35] Caro C.; Guzzi C.; Moral-Sanchez I.; Urbano-Gamez J. D.; Beltran A. M.; Garcia-Martin M. L. Smart Design of ZnFe and ZnFe@ Fe Nanoparticles for MRI-tracked Magnetic Hyperthermia Therapy: Challenging Classical Theories of Nanoparticles Growth and Nanomagnetism. Adv. Healthcare Mater. 2024, 2304044 10.1002/adhm.202304044. 38303644

[ref36] Norek M.; Peters J. A. MRI contrast agents based on dysprosium or holmium. Prog. Nucl. Magn. Reson. Spectrosc. 2011, 59, 64–82. 10.1016/j.pnmrs.2010.08.002. 21600356

[ref37] Zhang X.; Blasiak B.; Marenco A. J.; Trudel S.; Tomanek B.; van Veggel F. C. J. M. Design and Regulation of NaHoF_4_ and NaDyF_4_ Nanoparticles for High-Field Magnetic Resonance Imaging. Chem. Mater. 2016, 28, 3060–3072. 10.1021/acs.chemmater.6b00264.

[ref38] Antwi-Baah R.; Wang Y.; Chen X.; Yu K. Metal-Based Nanoparticle Magnetic Resonance Imaging Contrast Agents: Classifications, Issues, and Countermeasures toward their Clinical Translation. Adv. Mater. Interfaces 2022, 9, 2101710 10.1002/admi.202101710.

[ref39] Gomez-Gonzalez E.; Gonzalez-Mancebo D.; Nunez N. O.; Caro C.; Garcia-Martin M. L.; Becerro A. I.; Ocana M. Lanthanide vanadate-based trimodal probes for near-infrared luminescent bioimaging, high-field magnetic resonance imaging, and X-ray computed tomography. J. Colloid Interface Sci. 2023, 646, 721–731. 10.1016/j.jcis.2023.05.078. 37229990

[ref40] Lee N.; Choi S. H.; Hyeon T. Nano-Sized CT Contrast Agents. Adv. Mater. 2013, 25, 2641–2660. 10.1002/adma.201300081. 23553799

