# Supplementary material for: Nanoparticulated Bimodal Contrast Agent for Ultra-High-Field Magnetic Resonance Imaging and Spectral X-ray Computed Tomography
Source: Inorg Chem. 2024 May 29;63(23):10648–56. doi: 10.1021/acs.inorgchem.4c01114 (PMC11167642; doi:10.1021/acs.inorgchem.4c01114)
Supplement: Supplementary file 1 — ic4c01114_si_001.pdf [file ic4c01114_si_001.pdf]

Electronic Supplementary Information

Nanoparticulated Bimodal Contrast Agent for Ultra-High-Field Magnetic Resonance Imaging and Spectral X-ray Computed Tomography

Daniel González-Mancebo,[1] Ana Isabel Becerro,[1*] Carlos Caro,[2,3,4] Elisabet Gómez-González,[1] María Luisa García-Martín,[2,3,4] Manuel Ocaña[1]

[1]Instituto de Ciencia de Materiales de Sevilla (CSIC-US), c/Américo Vespucio, 49, 41092 Seville, Spain.

[2]Biomedical Magnetic Resonance Laboratory-BMRL, Andalusian Public Foundation Progress and Health-FPS, 41092 Seville, Spain

[3]Instituto de Investigación Biomédica de Málaga y Plataforma en Nanomedicina – IBIMA Plataforma BIONAND Málaga 29590, Spain

[4]CIBER-BBN, ISCIII

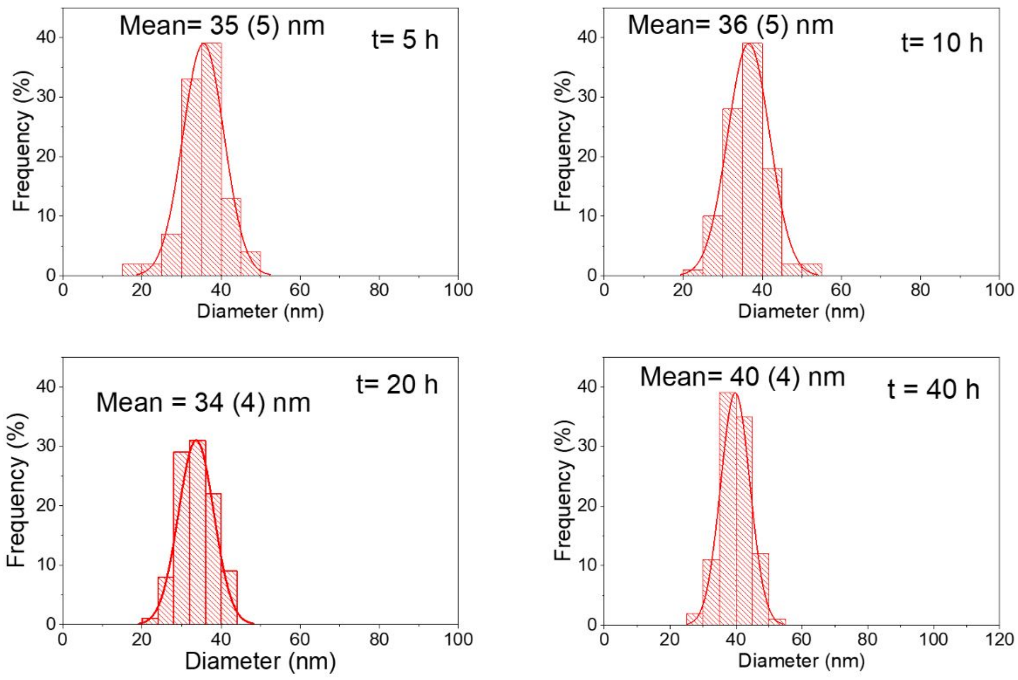

Figure S1: Size histograms obtained from TEM micrographs of the NPs synthesized at different reaction times using nominal Ba/(Ba+Dy) = 0.75.

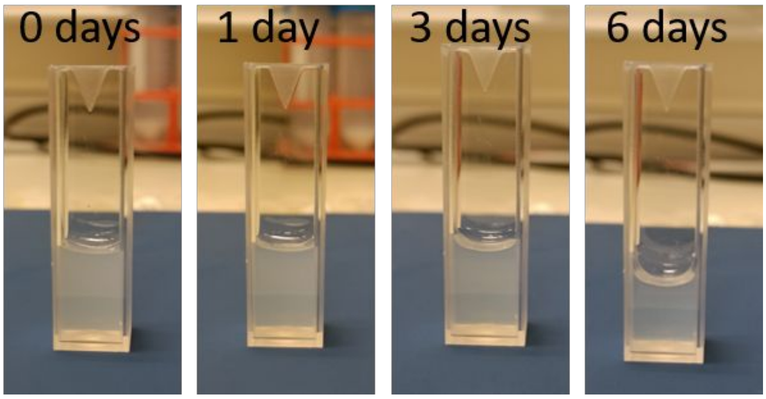

Figure S2: Photographs of Ba51Dy49 NPs suspensions taken after different periods of time at rest.

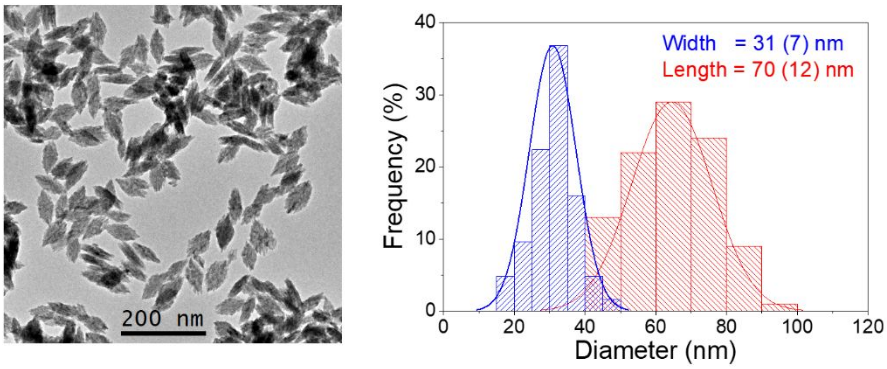

Figure S3: TEM micrograph and size histograms of DyF_3 NPs synthesised as follows: $Dy(acac)_3$ (0.067 M) was dispersed in glycerol (6 mL) with magnetic stirring at 80 °C for 3 hours. After cooling down, 205.5 µL of $[BMIM]BF_4$ (0.55 M) were added with magnetic stirring for 3 minutes at room temperature to favor homogenization. The resulting dispersion was transferred to a tightly closed Teflon test tube and heated for 20 hours in an oven preheated at 120 ºC. After cooling down, the dispersion was washed twice with ethanol and once with distilled water.
